# Stereolithography of Semiconductor Silver and Acrylic-Based Nanocomposites

**DOI:** 10.3390/polym14235238

**Published:** 2022-12-01

**Authors:** Luisa M. Valencia, Miriam Herrera, María de la Mata, Jesús Hernández-Saz, Ismael Romero-Ocaña, Francisco J. Delgado, Javier Benito, Sergio I. Molina

**Affiliations:** 1 Departamento de Ciencia de los Materiales e Ingeniería Metalúrgica y Química Inorgánica, IMEYMAT, Facultad de Ciencias, Universidad de Cádiz, Campus Río San Pedro, s/n, 11510 Cádiz, Spain; 2 Departamento de Ingeniería y Ciencia de los Materiales y del Transporte, Universidad de Sevilla, Avda. Camino de los Descubrimientos s/n, 41092 Sevilla, Spain

**Keywords:** stereolithography, polymer-based nanocomposites, (semi)conducting polymeric nanocomposites, acrylic resin, silver nanoparticles, semiconductor

## Abstract

Polymer nanocomposites (PNCs) attract the attention of researchers and industry because of their potential properties in widespread fields. Specifically, electrically conductive and semiconductor PNCs are gaining interest as promising materials for biomedical, optoelectronic and sensing applications, among others. Here, metallic nanoparticles (NPs) are extensively used as nanoadditives to increase the electrical conductivity of mere acrylic resin. As the in situ formation of metallic NPs within the acrylic matrix is hindered by the solubility of the NP precursors, we propose a method to increase the density of Ag NPs by using different intermediate solvents, allowing preparation of Ag/acrylic resin nanocomposites with improved electrical behaviour. We fabricated 3D structures using stereolithography (SLA) by dissolving different quantities of metal precursor (AgClO_4_) in methanol and in *N*,*N*-dimethylformamide (DMF) and adding these solutions to the acrylic resin. The high density of Ag NPs obtained notably increases the electrical conductivity of the nanocomposites, reaching the semiconductor regime. We analysed the effect of the auxiliary solvents during the printing process and the implications on the mechanical properties and the degree of cure of the fabricated nanocomposites. The good quality of the materials prepared by this method turn these nanocomposites into promising candidates for electronic applications.

## 1. Introduction

Over the past few years, the synthesis of (semi)conducting polymer nanocomposites (PNCs) has undergone in-depth investigations with a careful selection of polymer matrixes and nanofillers [1,2,3]. With this aim, conductive nanomaterials are often dispersed in insulating polymer matrixes to fabricate (semi)conducting PNCs, which are promising resources for biomedical, optoelectronic and sensing applications [4,5,6]. Within this context, the incorporation of metallic nanoparticles (NPs) as fillers is an emerging approach to enhance the performance of (semi)conducting PNCs [7,8], since metallic NPs offer appealing physicochemical properties due to their high surface area to volume ratio.

In particular, Ag NPs are increasingly used as nanofillers in PNCs because of their excellent conductivity and antibacterial properties [9,10,11,12]. Ag NPs are commonly prepared by chemical reduction in an organic or aqueous medium containing stabilizers [13,14] and then are added to a polymer solution to obtain the Ag/polymer composites [15,16,17]. As an interesting alternative, Sangermano et al. proposed the in situ synthesis of Ag-epoxy [18] and Au-acrylic [19] nanocomposites, where a radical photoinitiator can simultaneously form the metallic NPs as well as generate reactive species for crosslinking polymerization upon UV irradiation. 

Different methods have been recently proposed to obtain Ag-PNCs using relevant additive manufacturing (AM) techniques. The main advantage of these methods is the in situ photoreduction of the Ag precursor during the printing process while the photopolymerization of the organic polymers takes place. Fantino et al. [20] reported the preparation of conductive 3D nanocomposites by coupling digital light processing (DLP) technology with the photoreduction of Ag precursors, generating Ag NPs during the UV post-curing process after the DLP. These authors also proposed an alternative thermal treatment to induce the formation of Ag NPs after the DLP process [21]. In situ approaches have also been considered in other AM technologies, such as in stereolithography (SLA). Customized SLA equipment has been proposed to fabricate acrylic-based structures with Ag-patterned surfaces by modifying the laser settings during the printing process, being suitable for resistive switching devices and bacteria proliferation control [22,23]. Additionally, Scancialepore et al. [24] reported the fabrication of 3D printed pieces with Ag NPs using low Ag precursor contents (Ag acetate), obtaining only a slight decrease in the electrical resistivity, in the same order of magnitude as that of pristine resin.

In a previous paper, the feasibility of fabricating nanocomposites based on acrylic photocurable formulations containing Ag NPs in situ generated by UV-induced reduction of AgNO_3_ and AgClO_4_ during the SLA printing process was proposed by the authors [25]. This approach involves the simultaneous polymerization of the acrylic resin and the reduction of Ag ions to metallic Ag by means of the UV laser action of an SLA printer. Although the electrical resistivity of the PNCs obtained was reduced four orders of magnitude, it still corresponded to insulator materials rather than to (semi)conductor materials, meaning that the amount of Ag NPs was not adequate to achieve the desired electrical conductivity. The solubility of the Ag precursors in the resin prevents the addition of more than 3 wt% of AgClO_4_ to obtain the nanocomposites. In the present paper, we propose an approach to overcome such limitations, aiming to increase the density of Ag NPs by using an intermediate solvent to assist the salt dissolution, rendering higher amounts of precursor dispersed in the resin. Solvents like water, ethanol, methanol, and *N,N*-dimethylformamide (DMF) have been extensively used for the synthesis of PNCs in the literature, to dissolve and help in the dispersion of Ag NPs [15,16,26,27,28,29]. In particular, we evaluate the effect of using methanol and DMF to increase the amount of AgClO_4_ that can be dispersed in an acrylic resin used in SLA, to improve the electrical conductivity of Ag nanocomposites. The presence of the external solvent can affect the mechanical properties of the material due to pore formation during evaporation. The effect of the nature of the external solvents on the structural and mechanical properties of the resulting nanocomposites is also studied and discussed.

## 2. Materials and Methods

### 2.1. Materials

Clear photopolymer standard resin (a mixture of proprietary acrylic monomers and oligomers and phenylbis (2,4,6-trimethyl benzoyl)-phosphine oxide as photoinitiator) was purchased from XYZprinting, Inc. (XYZprinting, New Taipei City, Taiwan). Phenylbis (2,4,6-trimethyl benzoyl)-phosphine oxide and silver perchlorate (AgClO_4_) were purchased from Alfa Aesar (Thermo Fisher, Kendal, Germany). Methanol, *N,N*-dimethylformamide (DMF), and isopropanol (IPA) were purchased from Scharlau (Scharlab, Sentmenat, Spain). All products were used as received.

### 2.2. Sample Preparation 

Two solvents, methanol and DMF, were used to disperse different quantities of AgClO_4_ (5, 10, and 15 wt%) in an acrylic resin (1 mL solvent/20 mL acrylic resin) to fabricate the nanocomposites. Based on the results obtained in our previous work [25], 2 wt% photoinitiator (phenylbis (2,4,6-trimethyl benzoyl)-phosphine oxide) was added to the solutions to improve the degree of cure of the acrylic resin. An Ultrasonic Cleaner USC500T provided by VWR (VWR International, Radnor, PA, USA) and working at 45 kHz was used for the sonication processes (30 min). Solid specimens were obtained by two different techniques:

- Using a UV chamber with a light source of 405 nm and a power of 1.25 mW/cm^2^ (FormCure, Formlabs, Somerville, MA, USA)) for 90 min. Previous to that, some solutions were left in vacuum during 24 h to evaporate the solvent.
- Printing by SLA with Nobel 1.0 equipment, XYZprinting, Inc. (XYZprinting, New Taipei City, Taiwan)., using a 405 nm laser with an output power of 100 mW and a spot size that allows an XY resolution of 300 µm. All samples were printed with a layer height of 100 µm. Once printed, the samples were washed in IPA for several minutes. Post-processing of the samples was also performed inside a UV chamber with a light source of 405 nm and a power of 1.25 mW/cm^2^ (FormCure, Formlabs) for 60 min.

Flat discs with a thickness of 2 mm and diameter of 65 mm were fabricated for electrical and mechanical measurements. Moreover, complex cubic structures with hollows and curved parts (2 × 2 × 2 cm^3^) were also printed. 

### 2.3. Characterization

Surface and cross-section structural analyses were performed using a Scios 2 DualBeam (Thermo Fisher Scientific, Waltham, MA, USA) focused ion beam-scanning electron microscope (FIB-SEM) working at 30 kV ion aceleration voltage. This equipment was also used to obtain electron-transparent thin films of the nanocomposites for transmission electron microscopy (TEM) analyses. The thin films were cleaned at low voltage (2 kV). 

Samples were coated prior to FIB-SEM analyses with a layer of Au in a SCD 004 Sputter Coater (BAL-TEC, Balzers, Lichtenstein).

High angle annular dark field scanning (HAADF-S-) TEM and Energy-dispersive X-ray (EDX) measurements were performed using a Thermo Scientific TALOS F200S (Thermo Fisher Scientific, Waltham, MA, USA) working at 200 kV.

Shore D hardness was carried out using a Durometer (Sauter HB (&TI), Vitoria-Gasteiz, Spain) according to ASTM D2240. At least five measurements were performed on each test specimen; the results were averaged, and standard deviations were determined.

Differential scanning calorimetry (DSC) was used to determine the curing enthalpy of the different samples with a Q20 (TA Instruments, New Castle, DE, USA). DSC curves were obtained by performing a temperature sweep from room temperature (25 °C) to 320 °C at 10 °C/min under a nitrogen atmosphere. A subsequent cooling and heating sweep at 10 °C/min was performed to confirm the complete polymerization of the resin in the first sweep.

Electrical resistivity was measured following ASTM D257 using a Keithley 6517B electrometer (Keithley, Cleveland, OH, USA) with a voltage of 500 V. At least three measurements were performed for each composite. Results were averaged, with standard deviations presented as error bars. 

## 3. Results

### 3.1. Fabrication of Nanocomposites: Photopolymerization in UV Chamber vs. SLA Printer

**Figure 1:**
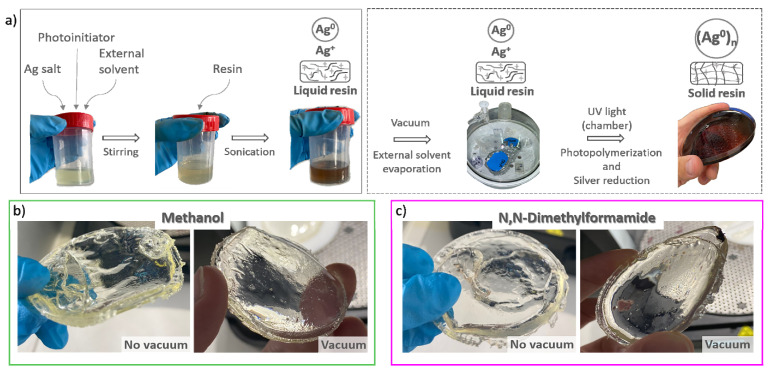
(**a**) Scheme representation of the preparation method of the 3D nanocomposites using a UV chamber; solid disks obtained from the solutions of acrylic resin containing methanol (**b**) and DMF (**c**) without (left) and with (right) the vacuum step added to the synthesis.

For comparative purposes, nanocomposites obtained by a conventional procedure, using a UV curing chamber for the photopolymerization (schematized in Figure 1a), were manufactured. Initially, the formulations were prepared by dissolving the desired concentrations of AgClO_4_ (including a reference formulation with no AgClO_4_) with a 2 wt% of photoinitiator in the two solvents considered, methanol and DMF, stirring the mixture to help the dissolution. After that, the mixture was added to the acrylic resin, and the new solution was sonicated to disperse and homogenize the solution. Once the formulations were prepared, they were poured into different molds and cured using the UV chamber to obtain the solid pieces. The photo-reactive process started by UV irradiation, triggering the formation of transient radical species able to initiate the radical polymerization of acrylic monomers to solidify the resin, while also initiating the reduction of Ag^+^ to Ag^0^ to create the Ag NPs [30,31,32,33]. Left images in Figure 1b,c show examples of solid structures made from the reference formulation with no AgClO_4_ obtained following this approach. As it can be observed, the fabricated pieces present distorted geometries due to the material shrinkage, along with large pores created as a consequence of the evaporation of the solvents remaining in the solutions. In order to avoid the formation of pores, an additional step was introduced in the procedure, prior to the photopolymerization of the composites, consisting of the removal of methanol and DMF solvents by vacuum exposure for 24 h. In this step, the pieces obtained show smoother surfaces, with no pores visible with the naked eye (right images in Figure 1b,c). Removal of part of the solvents may produce partial precipitation of the AgClO_4_ dispersed in the resin, according to the solubility limits found in our previous work [25]. However, no precipitates were visually detected after vacuum exposure in the liquid resin in any of the mixtures, likely due to a good dispersion of the precursor in the resin. While local and smaller AgClO_4_ precipitates may form in the resin, some of them could still react to give place to Ag NPs. Regarding the geometry of the pieces, they show shapes deviating from the expected shape due to internal stress during the photopolymerization. The conventional photopolymerization reactions induced by UV light are currently restricted to small surface volumes (thin layers, <50 µm) where the light penetration is large enough to ensure a fast and efficient curing [34]. For thicker samples, such as those presented in Figure 1, the light penetration is not deep enough to achieve an efficient and uniform polymerization process of the complete material volume, resulting in different curing rates over the sample. The first layer polymerizes faster due to the larger UV light intensity; however, for deeper layers, the curing process slows down, since the UV penetration is smaller, creating points of local stress in the material and non-uniform shrinkage of the solid piece. Additionally, shadow areas where UV light is blocked by the geometry of the mold also polymerize at slower rates, inducing additional tensions. The shrinkage of acrylic resins during photopolymerization has been previously reported by several authors in the literature [35,36,37,38], with different strategies to decrease the tensions during the polymerization, such as modifying the chemistry of the oligomers and monomers, the use of thiol-ene resins, or thinning the samples. Although thinning the samples may be beneficial in our case to achieve uniform curing throughout the sample, solid pieces with uniform thicknesses of 2 mm are required to perform electrical measurements according to ASTM D257 specifications. Additional steps involving changes in the chemistry of the oligomers and monomers of the commercial resin used would be needed to obtain appropriate uniform pieces for electrical measurements, which are out of the scope of the present study. 

**Figure 2:**
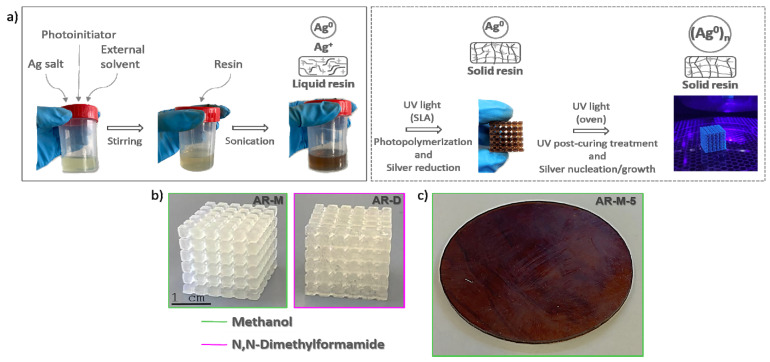
(**a**) Scheme depicting the process followed to prepare 3D printed nanocomposites using SLA (the box with continuous line includes the steps that are common with the method based in melds as explained above, and the dashed box shows the specific steps for the SLA method); (**b**) printed cubes of the solutions containing the mixture of unfilled resin with methanol (green) and DMF (purple); (**c**) disk printed by SLA of the solution containing methanol and 5 wt% AgClO_4_.

Regarding the fabrication of (semi)conducting nanocomposites by AM technologies, 3D structures were printed by SLA following the process disposed in Figure 2a. The external solvents, the photoinitiator, and the required amount of Ag precursor to fabricate 5, 10, and 15 wt% AgClO_4_ nanocomposites were mixed, stirred, and added to the acrylic resin, following the same procedure as with the pieces solidified in the UV chamber. These solutions were sonicated in order to homogenize the mixture and poured into the SLA tank, to carry out the simultaneous photopolymerization of the acrylic resin and the reduction of the Ag cations into metallic Ag NPs during the printing process. Subsequently, the printed pieces were post-cured inside the UV chamber to enhance the degree of cure of the nanocomposites. This approach allowed the successful printing of complex geometries (Figure 2b) with smooth surfaces and lacking large surface pores. The better quality of the printed pieces compared to those obtained using molds could be related to the intrinsic procedure of SLA printing. The selective photopolymerization of the liquid resin layer-by-layer by the UV beam during SLA allowed most of the solvents to remain in the printer tank instead of being trapped within the acrylic resin as in the UV chamber method. In addition, since the acrylic resin was selectively cured, by using the appropriate printing parameters, the curing process was controlled, and no stresses appeared during the photopolymerization, allowing precise geometries.

It should be mentioned that, while nanocomposites with AgClO_4_ concentrations up to 10 wt% print successfully, higher precursor concentrations hinder the polymer curing, degrading the quality of the pieces. This effect was observed regardless of the solvent (methanol or DMF) when printing 15 wt% AgClO_4_ 3D nanocomposites using the same amount of solvent as that used for the 5 and 10 wt% AgClO_4_ 3D nanocomposites. Consequently, the poor quality of the printed structures for 15 wt% AgClO_4_ is likely related to the large amount of Ag precursor used. The high AgClO_4_ considered may affect the polymerization of the resin, since the Ag reduction and the resin curing are triggered by the same photoinitiator (even with the addition of an extra amount of photoinitiator). Ag NPs present a strong absorption band around 400 nm, which overlaps the absorption band of the photoinitiator of the acrylic resin (see Figure S1, Supporting Information). This large absorption by the Ag NPs could reduce the activity of the photoinitiator, producing a final degree of cure of the resin smaller than expected. Despite this negative result for the higher AgClO_4_ content studied, our results evidence the benefits of external solvents assisting the dispersion of the Ag precursor in the matrix resin for the successful printing of 3D pieces. However, attention should be paid to the effect of the solvents and the AgClO_4_ on the microstructure and on the electrical and mechanical properties of the fabricated nanocomposites; this will be analyzed next. For clarity, the materials studied are labelled with letters indicating the solvent nature and numbers indicating the precursor concentration, as shown in Table 1.

### 3.2. Morphological and Structural Characterization

**Figure 3:**
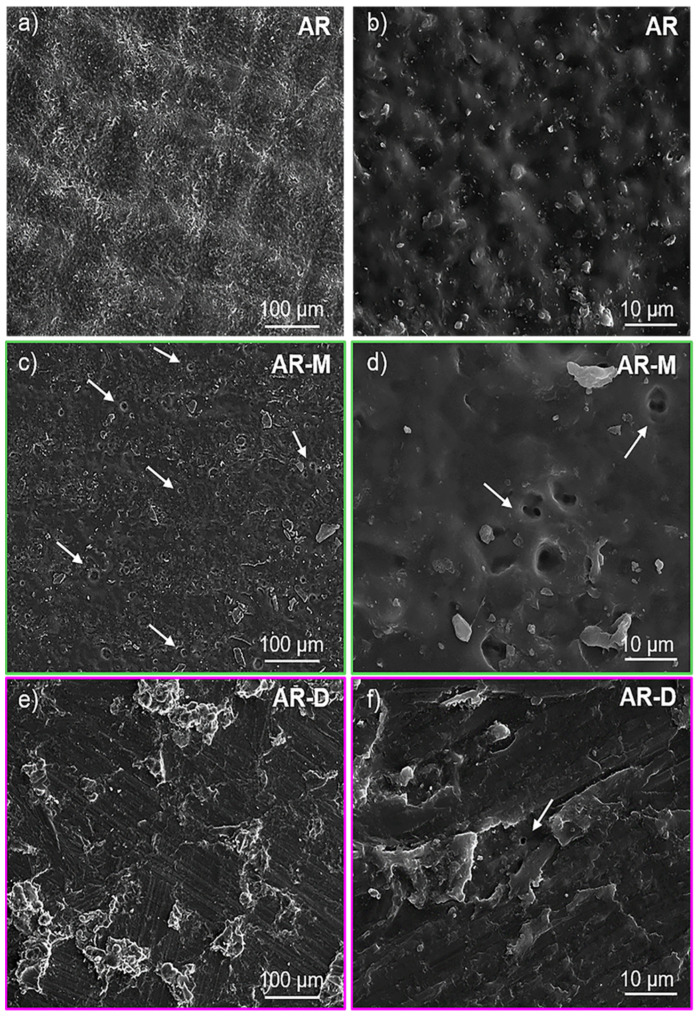
SEM images of samples AR (**a**,**b**), AR-M (**c**,**d**), and AR-D (**e**,**f**) obtained at lower (left) and higher (right) magnifications.

We evaluated the effect of adding external solvents on the structural properties of the acrylic matrix, in particular the possible formation of surface micropores resulting from the solvent evaporation. SEM analyses of samples AR-M and AR-D were carried out and compared to the reference specimen, AR, cured without any external solvent. Figure 3 shows SEM images of the different samples, obtained at two different magnifications. In the case of the pristine resin, Figure 3a,b shows the rough but homogeneous surface from sample AR, with no porosity. However, samples AR-M and AR-D (Figure 3c–f) exhibit some micropores (pointed with arrows) throughout the surface of the acrylic matrix, with a larger density in AR-M. These micropores are likely due to the evaporation of any residue from the solvents, presumably trapped in the polymeric matrix while printing the material, during the post-curing treatment. The UV post-curing process is exothermic and can lead to areas with some increment of the temperature, reaching the necessary temperature to evaporate the solvent. The higher surface porosity of sample AR-M is due to the lower boiling point of methanol (65 °C) in comparison to DMF (153 °C), which causes the solvent to evaporate at lower temperatures. 

**Figure 4:**
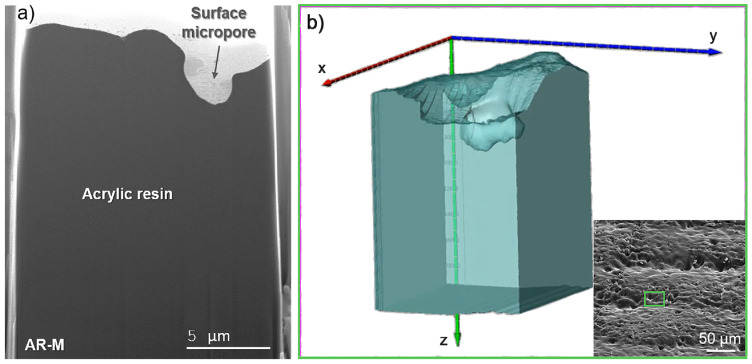
(**a**) SEM image and (**b**) slice and view FIB-SEM tomography of sample AR-M. The inset displays an SEM image with a green rectangle pointing out the area studied.

Due to the results obtained by SEM and in order to explore the presence of internal pores within the cured acrylic resin, slice and view FIB-SEM tomography were conducted, and the results for sample AR-M can be found in Figure 4. Figure 4a shows an SEM image of one of the cross-section slices obtained, showing the interior of the material, and Figure 4b displays a reconstruction of the 3D volume obtained in the area marked with a green rectangle in the inset image. In this analysis, the surface characteristics observed by SEM were corroborated, showing the presence of surface micropores such as those exhibited in Figure 3. However, it should be highlighted that no internal pores were found throughout the acrylic matrix in the different analyses carried out. The solvents remaining in the solid matrix could be either a negligible amount or unable to evaporate due to being trapped and mixed with the solid resin. This result is very advantageous in relation to the mechanical properties of the materials, as micropores may weaken the nanocomposites, reducing their tensile strength. 

**Figure 5:**
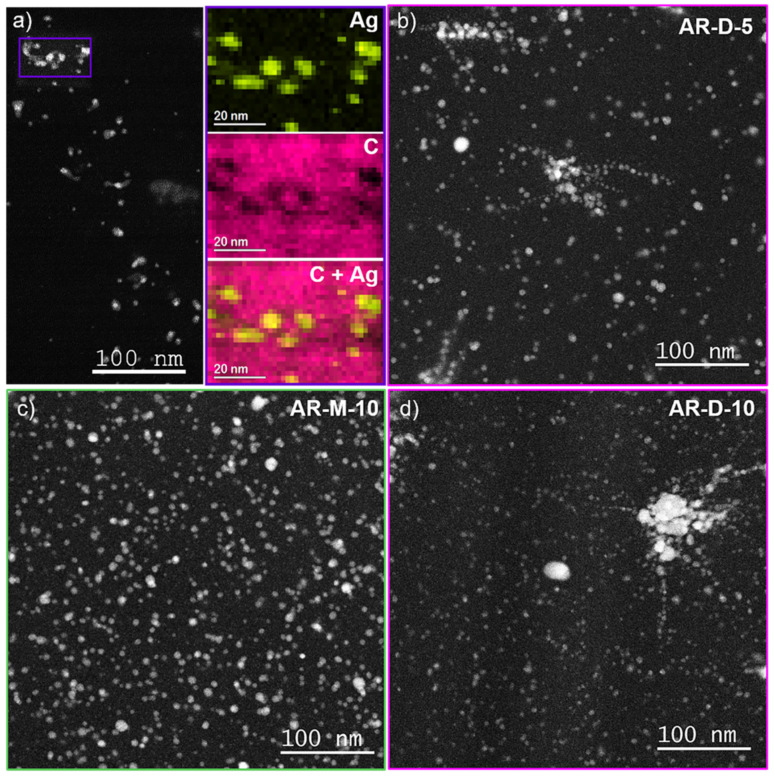
(**a**) HAADF-STEM image of nanocomposite AR-D-5 and EDX map of the squared area showing the Ag signal from the nanoparticles (yellow) and the C signal from the acrylic resin (pink); HAADF-STEM images of 3D printed objects containing acrylic resin, external solvent, and different concentrations of silver precursor: (**b**) AR-D-5; (**c**) AR-M-10, and (**d**) AR-D-10.

In order to analyze the Ag NPs that are expected to form within the acrylic matrix during the resin photopolymerization due to the addition of the Ag precursor AgClO_4_, TEM analyses of the nanocomposites fabricated were carried out. Figure 5a,b displays HAADF-STEM images of sample AR-D-5. In HAADF-STEM, the contrast of the images is related to the average Z number of the material; therefore, heavier atoms produce brighter contrast in the images. Accordingly, the brighter regions in the images are expected to correspond to the presence of Ag NPs. We further confirmed the nature of the NPs by EDX measurements, allowing mapping of the chemical composition of the nanocomposites (Figure 5a). Here, C and Ag signals are shown in pink and yellow, respectively. These results confirm the in situ formation of Ag NPs during the photopolymerization of the acrylic resin. A large density of Ag NPs was found in the samples with 5% AgClO_4_, which is even larger for the PNC with 10% AgClO_4_, as can be observed in Figure 5c,d for samples AR-M-10 and AR-D-10, respectively. In relation to the effect of the different solvents used, for DMF areas with particle aggregation were noticed, as can be seen in Figure 5b,d. This could be related to a non-uniform distribution of the solution containing DMF and Ag precursor in the liquid acrylic resin, since the viscosity of DMF is larger than that of methanol, which may make difficult an effective dispersion during the sonication process. In any case, the large density of Ag NPs is promising for the enhancement of the electrical properties of the nanocomposites, as analyzed below.

### 3.3. Shore D Hardness Measurements and Analysis of the Degree of Cure

Shore D hardness was measured for each 3D-printed nanocomposite (summarized in Table 2) in order to study the influence of the solvents and the addition of AgClO_4_ in the mechanical behavior of the fabricated nanocomposites. Regarding the use of intermediate solvents, hardness values of 66.4 ± 2.6 and 71.6 ± 3.1 were measured for samples AR-M and AR-D, respectively. There was a slight reduction of the hardness of around 15% in the case of AR-M and 8% for AR-D compared to the reference sample without solvent, AR (measured hardness of 78.2 ± 2.4). This could be the result of traces of solvent that remained trapped in the acrylic matrix after the post-curing process, resulting in a degree of cure slightly lower than expected. The presence of surface micropores observed by SEM could also have some effect in this regard. However, a material with 66.4 HBD, as is the case of AR-M, is still an extra-hard material according to ASTM D2240.

**Figure 6:**
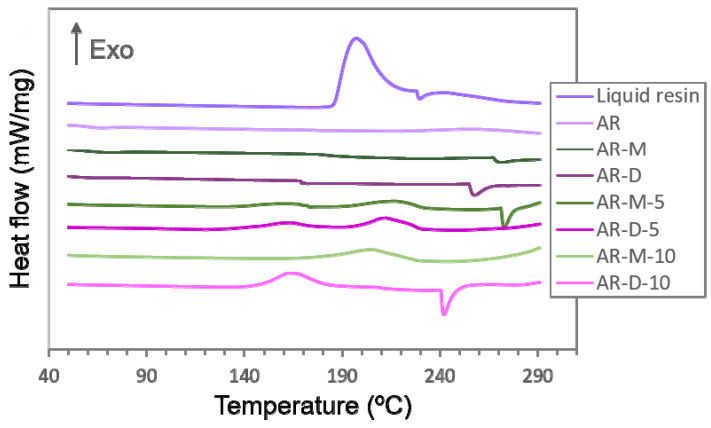
DSC thermograms of the liquid resin and the Ag nanocomposites.

The effect of the Ag precursor in the acrylic resin is much more pronounced, softening the material remarkably. Note that the addition of 5 wt% AgClO_4_ to the polymer matrix already reduces the hardness by around 30–40%, whereas by adding 10 wt% AgClO_4_, hardness is reduced by almost 50% compared to the reference material, AR. This fact could be explained by the competition for the photoinitiator, which, as explained before, is involved in two simultaneous processes, the formation of the Ag NPs and the polymerization of the acrylic resin. The addition of a high concentration of Ag precursor consumes a great deal of the photoinitiator to synthesize the Ag NPs, which hinders the photopolymerization of the acrylic resin, affecting the final degree of cure of the material [25]. Thermal analyses of the nanocomposites by differential scanning calorimetry (DSC) were carried out in order to test the influence of the AgClO_4_ on the photopolymerization of the acrylic resin. Figure 6 shows the DSC curves of the liquid resin and the printed samples. The liquid resin presented an exothermic peak at 185–220 ℃, corresponding to the cure enthalpy (Δ*H_cure_*). In the case of the 3D objects, the values obtained from the DSC sweeps correspond to the residuary cure enthalpy (*ΔH_residuary_*). Hence, the degree of cure can be calculated as follows:

(1) $$Degree\;of\;cure\;\left(\%\right)=\left(1-\frac{\Delta H_{residuary}}{\Delta H_{cure}}\right)\times 100$$

Following Equation (1), we calculated the degree of cure of the nanocomposites for the data obtained in the DSC curves. As can be observed in Figure 6, the samples without Ag fillers (AR, AR-M and AR-D) do not show any noticeable band, meaning that a full degree of cure has been achieved. However, several bands can be observed for the samples containing Ag NPs, indicating the presence of unreacted monomer in the resin. For samples AR-M-5, AR-D-5, AR-M-10, and AR-D-10, similar results were obtained, with a degree of cure of approximately 75%. This is likely related to the higher density of Ag NPs that would require a larger amount of photoinitiator to be synthetized. Even when we readjusted the amount of photoinitiator in order to compensate for its consumption during the reduction of the Ag precursor (by adding an extra 2 wt% of photoinitiator), complete photopolymerization was still not achieved. In the literature, it has been reported that a shortage of photoinitiator in Cu/Ag nanocomposites prepared by SLA also showed a worsening of the mechanical properties of the material, becoming more brittle than the acrylic reference sample [39]. Nonetheless, the hardness values of the nanocomposites fabricated in this work are in the range of 30–50 HBD, which corresponds to the transition between medium-hard and hard materials in the shore D hardness scale, being suitable materials for technological applications.

### 3.4. Electrical Properties of the Fabricated Nanocomposites

**Figure 7:**
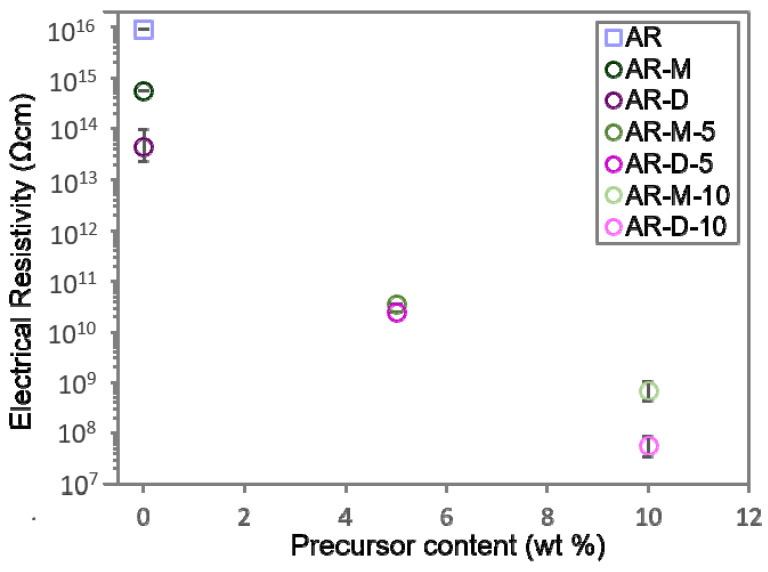
Electrical resistivity for the nanocomposites containing 0–10 wt% AgClO_4_ and methanol/DMF solvents.

Electrical measurements were carried out for the fabricated materials to analyse possible variations in the volumetric resistivity resulting from the formation of Ag NPs within the acrylic resin. Figure 7 shows the results obtained for the samples studied. The electrical resistivity of the pristine acrylic resin (AR) is in the range of 10^16^ Ωcm, meaning a strong insulating behavior. It was found that the electrical resistivity decreases by one to two orders of magnitude compared to the reference sample through the addition of methanol-DMF. As mentioned previously, traces of solvent may remain trapped in the acrylic matrix (see Section 2 of the Supporting Information), which might increase the electron mobility inside the acrylic matrix, improving the electrical performance of the material [40]. 

Regarding the nanocomposites containing Ag NPs, the electrical resistivity decreases as the AgClO_4_ concentration incresases, meaning an improvement in the electrical properties of the material, as expected due to the high conductivity of Ag NPs. In absence of an external solvent, the acrylic resin is able to disolve up to 3 wt% AgClO_4_, reducing by four orders of magnitude the electrical resistivity regarding the pristine resin [25]. In this work, the use of methanol and DMF as intermediate solvents has allowed increasing the amount of AgClO_4_ to 5 and 10 wt%, increasing the amount of Ag NPs formed in the nanocomposites (see Table S2, Supporting Information). In particular, a reduction of the electrical resistivity of around six orders of magnitude was obtained when adding 5 wt% AgClO_4_ to the acrylic matrix for both solvents. However, this value still corresponds to insulators rather than to (semi)conductor materials due to the remarkably large electrical resistivity of the pristine resin (AR). Interestingly, a further decrease of around eight orders of magnitude regarding AR was obtained when the Ag precursor content was increased to 10 wt%, reaching resistivity values of 10^8^ Ωcm for AR-M-10, which is in the limit between insulator materials and semiconductors, and 10^7^ Ωcm for AR-D-10, already lying within the semiconductor range. This improvement in the electrical properties is in line with other nanocomposites found in the literature. For instance, Fantino et al. [20] reported fabrication using DLP of 3D conductive nanocomposites composed of polyethylene glycol diacrylate (PEGDA) as a polymer matrix and Ag NPs as metallic nanofillers. They reached the conductive threshold by adding only 5 wt% of AgNO_3_, since the initial electrical resistivity of the pristine matrix is only of 10^8^ Ωcm. Our results evidence the contribution of the intermediate solvents to further decrease the electrical resistivity in SLA Ag-containing nanocomposites. This opens the possibility to obtain electrically conductive pieces by SLA, by using as primary materials SLA resins with a lower initial electrical resistivity. Promising analyses in this area are in progress.

## 4. Conclusions

In this work, we evaluated the use of intermediate solvents to improve the electrical conductivity of Ag-containing SLA nanocomposites. For this, the solvents considered were methanol and DMF, used to increase the amount of AgClO_4_ that can be dispersed in an acrylic resin. We established a procedure where nanocomposites containing a large density of Ag NPs uniformly distributed through the acrylic resin could be successfully manufactured using SLA. Our results show that the evaporation of the solvents during the post-curing treatment gives place to some surface micropores, mostly in the case of methanol. However, pores were not found in the interior of the materials, which is beneficial for the mechanical properties of the composites. A decrease in the hardness values of around 8–15% in comparison to pristine resin was found upon the addition of solvents, likely due to a lower degree of cure associated with small amounts of solvents that remained trapped in the resin. Although hardness was further decreased with the addition of AgClO_4_ (to 50% of its initial value for 10 wt% AgClO_4_), the values obtained still correspond to reasonably hard materials, suitable for technological applications. Remarkably, a reduction in the electrical resistivity of around eight orders of magnitude was achieved for the nanocomposite prepared with DMF and containing 10 wt% AgClO_4_, thus becoming a semiconductor polymer nanocomposite. The method proposed in this work enables the fabrication of 3D complex and light pieces with promising electrical properties, which could be used as a good alternative in the electronics market.

## Figures and Tables

**Table 1:** Prepared nanocomposites (^♦^ AR: acrylic resin).

Composite	External Solvent	AgClO_4_ Content (wt%)
**AR ^♦^**	--	--
**AR-M**	Methanol	--
**AR-D**	*N,N*-Dimethylformamide	--
**AR-M-5**	Methanol	5
**AR-D-5**	*N,N*-Dimethylformamide	5
**AR-M-10**	Methanol	10
**AR-D-10**	*N,N*-Dimethylformamide	10

**Table 2:** Shore D hardness values of AR and the nanocomposites printed in this work.

Composite	Shore D Hardness (HBD)	Standard Deviation
**AR**	78.2	±2.4
**AR-M**	66.4	±2.6
**AR-D**	71.6	±3.1
**AR-M-5**	38.0	±1.6
**AR-D-5**	50.0	±2.5
**AR-M-10**	30.2	±2.9
**AR-D-10**	40.6	±3.2

## Data Availability

Not applicable.

## Supplementary Materials

The following supporting information can be downloaded at: https://www.mdpi.com/article/10.3390/polym14235238/s1, Figure S1: UV-Vis spectra of (a) sample AR and AgClO_4_ diluted in methanol and (b) samples AR, AR-M-5, AR-M-10 and AR-M-15; (c) spectra in (b) normalized taking the band from AR as a reference. Figure S2: (a) TGA and (b) DTG curves of the nanocomposites containing methanol/DMF solvents and 0–10 wt% AgClO_4_. Table S1: Amount of solvent present in the nanocomposites deducted from the TGA analysis. Table S2: Weight percentage of Ag in the printed nanocomposites.
