# Supplementary material for: Stereolithography of Semiconductor Silver and Acrylic-Based Nanocomposites
Source: Polymers (Basel). 2022 Dec 1;14(23):5238. doi: 10.3390/polym14235238 (PMC9736969; doi:10.3390/polym14235238)
Supplement: Supplementary file 1 [file polymers-14-05238-s001.zip › polymers-2024812-supplementary.pdf]

Supporting Information

Stereolithography of Semiconductor Silver and Acrylic-based Nanocomposites

Luisa M. Valencia [1,*], Miriam Herrera [1], María de la Mata [1], Jesús Hernández-Saz [2], Ismael Romero-Ocaña [1], Francisco J. Delgado [1], Javier Benito [1] and Sergio I. Molina [1]

[1] Departamento de Ciencia de los Materiales e Ingeniería Metalúrgica y Química Inorgánica, IMEYMAT, Facultad de Ciencias, Universidad de Cádiz, Campus Río San Pedro, s/n, 11510 Puerto Real (Cádiz), Spain

[2] Departamento de Ingeniería y Ciencia de los Materiales y del Transporte, Universidad de Sevilla, Avda. Camino de los Descubrimientos s/n, 41092 Sevilla, Spain

Index

1. UV-Vis spectrophotometry measurements of the Ag nanocomposites

   UV-Vis spectra of samples AR, ARM-5, ARM-10, ARM-15 and $AgClO_4$: Figure S1

2. Thermal Characterization

   TGA analysis of the nanocomposites: Table S1 and Figure S2

3. Chemical analysis by ICP

   Wt% of Ag in the nanocomposites calculated from ICP: Table S2

1. UV-Vis spectrophotometry measurements of the Ag nanocomposites

Figures S1b-c show the UV-Vis spectra of the different Ag nanocomposites printed using methanol as the external solvent. Since the absorption band of the acrylic resin lies in the same spectral region as the Ag NPs (Figure S1b), the spectra obtained for the nanocomposites have been normalized taking as a reference this band from AR (Figure S1c).

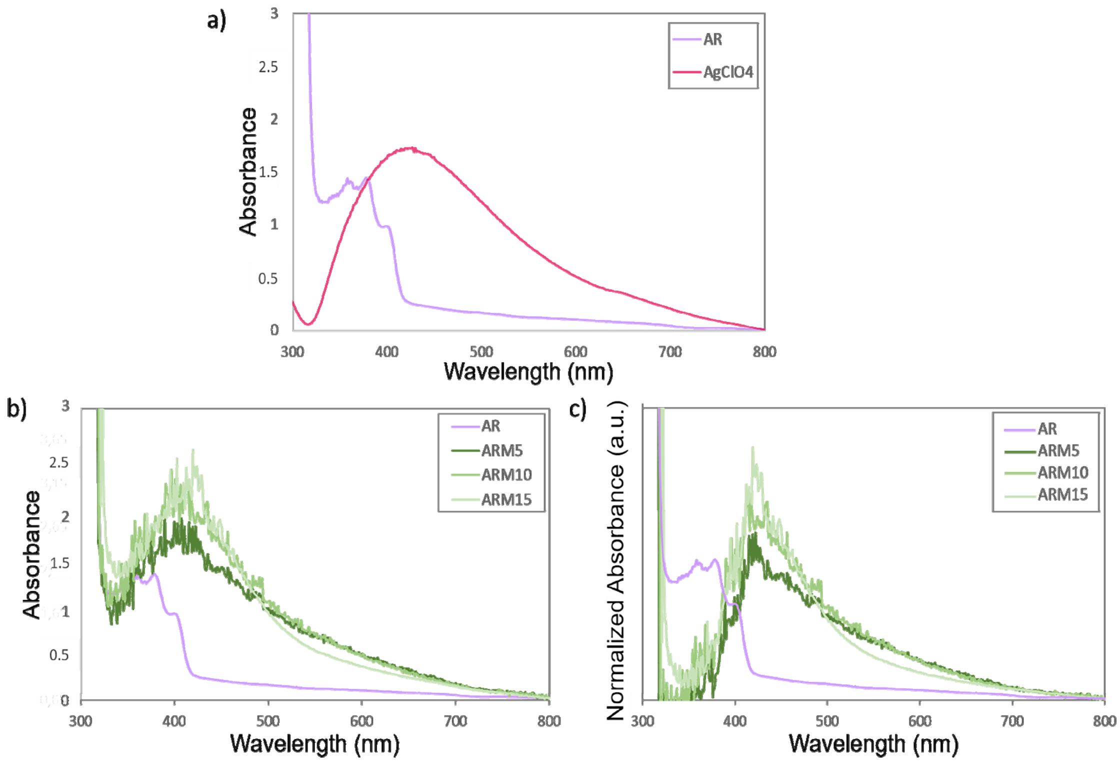

Figure S1. UV-Vis spectra of (**a**) sample AR and $AgClO_4$ diluted in methanol and (**b**) samples AR, AR-M-5, AR-M-10 and AR-M-15; (**c**) spectra in b) normalized taking the band from AR as a reference.

2. Thermal Characterization

Thermal experiments by thermogravimetric analysis (TGA) have been carried out to determine the amount of solvent in each formulation studied. The experimental conditions were as follows:

Experiment temperature from 30 °C to 600 °C. Ramp of 10 °C/min. Previous stabilization at 30 °C for 10 min. Nitrogen (N_2) inert atmosphere.

Table S1 shows the different amounts calculated according to the components of the formulation (AR; DMF (D); methanol (M) and Ag).

Table S1. Amount of solvent present in the nanocomposites deducted from the TGA analysis.

Composite	Initial mass TGA (mg)	Final mass TGA TGA (mg)	Solvent mass in residual mass (mg)	% Solvent mass on printed part
**AR**	15	0.7676	----	----
**AR-M**	17.224	1.0418	0.0068	0.039
**AR-D**	11.207	0.5671	0.138	1.23
**AR-M-5**	17.685	2.3290	0.0010	0.041
**AR-D-5**	16.422	2.4878	0.044	1.8
**AR-M-10**	14.867	2.7000	0.0011	0.034
**AR-D-10**	15.461	2.3481	0.038	1.59

As can be seen in Table S1, the mass quantities (mg) of the solvent for the different printed parts are very low. This suggests that the solvent is acting as a mere dispersant for the Ag NPs and does not interfere with the printed parts.

Figure S2 shows the graph corresponding to the TGA curves for the different samples studied. The behaviour for the different formulations is similar, there are slight differences due to the composition of the samples, but no significant changes are observed. In the graph corresponding to the % of weight derivative with respect to temperature (DTG), it can be seen that all samples have curves that are similar in overall shape. However, there are distinctive weight differences in the range between 450 °C to 600 °C (passive pyrolysis zone). This differences are due to the Ag loading contents. It can be observed that the highest percentage of mass loss occurs around 400 °C. This peak corresponds to the degradation of the resin (AR), the main compound present in the different formulations.

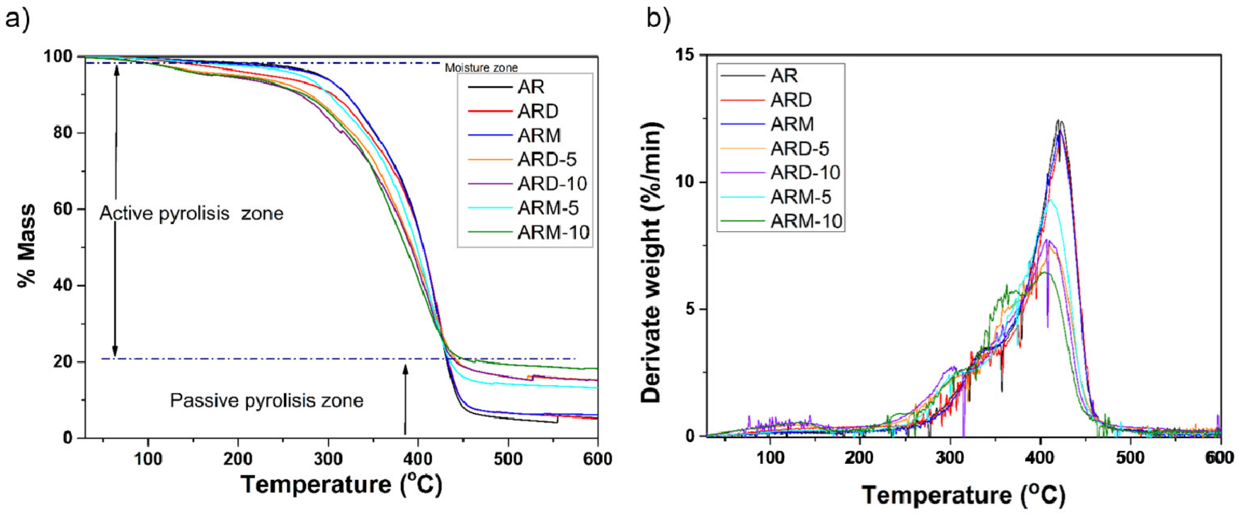

Figure S2. (**a**) TGA and (**b**) DTG curves of the nanocomposites containing methanol/DMF solvents and 0-10 wt% $AgClO_4$.

3. Chemical analysis by ICP

The weight percentage of Ag has been determined by ICP (Inductively Coupled Plasma). The printed samples have been studied. For this purpose, the samples were milled separately in a cryogenic mill. The powder obtained was then sieved through a 125 μm mesh screen. Before ICP measurement, an acid digestion was performed in triplicate on a 5-mg sample, weighed exactly and brought to 0.050 L with H_2O MQ. The results are shown in table S2 as the mean value of the three preparations together with the standard deviation. The ICP results show that samples with higher Ag precursor tend to concentrate a higher wt% of Ag NPs, regardless of the solvent.

Table S2. Weight percentage of Ag in the printed nanocomposites.

Composite	Ag (wt%)
**AR**	<0.0003*
**AR-M-5**	0.760 ± 0.050
**AR-D-5**	0.700 ± 0.050
**AR-M-10**	1.40 ± 0.10
**AR-D-10**	1.20 ± 0.10

* Result expressed as lower than that of the corresponding detection limit of the analytical method. Calculated as 3×Sb/m, where Sb is the standard deviation of the blank (n=10) and m is the slope of the calibration line, referred to the sample weight.

The wt% of Ag calculated by ICP differ from the theoretical calculations due to the fact that during the acid digestion part of the Ag remains in the resin which acts as a matrix and the Ag cannot be fully extracted. However, it is confirmed that the higher the amount of precursor, the higher the wt% of Ag in the printed samples.
